# Effects of Pyriproxyfen on Female Reproduction in the Common Cutworm, *Spodoptera litura* (F.) (Lepidoptera: Noctuidae)

**DOI:** 10.1371/journal.pone.0138171

**Published:** 2015-10-07

**Authors:** Qi Xu, Bin Tang, Qi Zou, Huizhen Zheng, Xiaojun Liu, Shigui Wang

**Affiliations:** Hangzhou Key Laboratory of Animal Adaptation and Evolution, School of Life and Environmental Sciences, Hangzhou Normal University, Hangzhou, 310036, Zhejiang, China; Institute of Zoology, Chinese Academy of Sciences, CHINA

## Abstract

The common cutworm, *Spodoptera litura*, is a rapidly reproducing pest of numerous agricultural ecosystems worldwide. The use of pesticides remains the primary means for controlling *S*. *litura*, despite their negative ecological impact and potential threat to human health. The use of exogenous hormone analogs may represent an alternative to insecticides. Juvenile hormones (JHs) play an important role in the reproductive systems of female insects, but the effects of pyriproxyfen, a JH analog, on reproduction in *S*. *litura* were poorly understood. In this paper, we topically treated the newly emerged females with 20, 60, or 100 μg of pyriproxyfen to determine its effects on reproduction. Then, we examined the expression of vitellogenin (*Vg*) and three hormone receptors, *USP*, *HR3*, and *EcR*, using quantitative reverse transcription and real-time polymerase chain reaction (qRT-PCR), and found that pyriproxyfen up-regulated the expression of *Vg*, *USP*, and *HR3*, whereas the expression of *EcR* was unaffected. An analysis of fecundity showed that the peak oviposition day, lifespan, and oviposition period were progressively shortened as the pyriproxyfen dosage increased. We also found that pyriproxyfen decreased egg laying amount, whereas the number of mature eggs that remained in the ovarioles of dead females increased as the pyriproxyfen dosage increased. We examined oocytes using transmission electron microscopy and found that treatment with 100 μg of pyriproxyfen increased the metabolism by increasing the amount of rough endoplasmic reticulum and mitochondria in the primary oocytes. Our results suggest that the topical application of pyriproxyfen on newly emerged females can efficiently reduce reproduction in *S*. *litura* and may represent an alternative to the use of insecticides for controlling the agricultural pest.

## Introduction

*Spodoptera litura* is a polyphagous and cosmopolitan pest of numerous types of agriculturally important plants, feeding on approximately 150 plant species from 40 families [1–4]. Although insecticides remain the most widely used approach for controlling *S*. *litura* [5], chemical pesticides can pollute the environment, and toxic levels of pesticides may accumulate in predators at the top of the food chain [6]. The extensive use of chemical pesticides has contributed to the development of resistance in a number of insect species [4], including *S*. *litura*, which has become more widely distributed in Asia in recent decades [7, 8].

Female reproductive characteristics in most insects can be influenced by various factors, including hormones and environmental changes [9, 10]. Insects were categorized into three groups based on the use of hormones in vitellogenin (*Vg*) transcriptional regulation. Lepidopterans belong to the third group, which requires juvenile hormones (JHs) and ecdysteroids during their reproductive development, however, a recent investigation showed that the *Vg* transcription regulation varies between species in this group [11]. In *S*. *litura*, supplemental growth regulators, such as pyriproxyfen (juvenile hormone analog, JHA), given to pupae greatly reduced its fertility, presumably by acting upon the endocrine system. Therefore, exogenous hormone analogs, such as juvenoids, may represent alternatives to traditional insecticides for controlling *S*. *litura* [10]. Although vitellogenesis is regulated by hormones in various ways [12, 13], a previous study of noctuid moths showed that JH is the primary regulator of vitellogenin expression in the fat body, the maintenance of ovariole patency, the uptake of Vg, and choriogenesis [14]. However, a more recent study indicated that both JH and ecdysone signaling are involved in vitellogenesis in various noctuid pest species, including *Spodoptera frugiperda* [15].

Applications of JHAs to decapitated or allatectomized females have been used to study the functions of hormones on *Vg* regulation. In Lepidopterans, specifically in *S*. *frugiperda* and *Pseudaletia unipunctata*, the levels of Vg production (mRNA or protein) in decapitated virgin females can be restored with JH or JHA treatments, such as methoprene or pyriproxyfen [15,16]. Similar results have been observed in *Apis mellifera* (hymenopterans) when decapitation was coupled with the application of JH-III [17]. Previous studies showed that treating *A*. *mellifera* females with low doses of JH or JHAs accelerated the initiation of *Vg* expression, whereas high doses inhibited *Vg* expression [18–20].

JHs and ecdysteroids are two important hormone families involved in regulating physiological events throughout the insect lifecycle, especially the reproductive system, including vitellogenesis and oocyte maturation [15, 17, 20, 21]. The JH and 20E signaling pathways and the cross-talk that occurs between them are complicated [22]. The nuclear hormone receptor, ultraspiracle (USP), and the ecdysone receptor (EcR) are candidate 20E receptors that form a heterodimeric protein complex that mediates the effects of ecdysone [22, 23]. Candidate downstream regulators include the 20E responsive hormone receptor 3 (HR3) [22]. Methoprene-tolerant (Met) is an important JH receptor, and its mutation alters juvenile hormone responses in insects [24–26].

In Lepidopterans, low dose JH or JHA treatments increase the number of eggs laid [14, 27, 28]. Studies on decapitated *Choristoneura fumiferana* and *C*. *rosaceana* showed that treating females with a JH analog, methoprene, increased egg production compared with control females [29], and a JH treatment alone was sufficient to restore choriogenesis. In a study of *Bicyclus anynana*, treatments with up to 100 μg of a JHA, pyriproxyfen, had no effects on reproductive parameters [30]. However, in female pupae of *S*. *litura*, treatments with a topical application of pyriproxyfen reduced egg production by decreasing the level of oviposition-stimulating factor, a 14 kDa protein, in the hemolymph [10]. Although there were some studies performed on the application of pyriproxyfen to larvae or pupae, no studies have been performed on the adult *S*. *litura* female. Additionally, the effects of pyriproxyfen on the female vitellogenesis still remain to be clarified when the pyriproxyfen is used on the newly emerged females.

In our current study, we examined the hormonal regulation of Vg expression in *S*. *litura* and found that JH and 20E were required for vitellogenesis and ovarian maturation. We measured levels of *Vg* mRNA in fat bodies of *S*. *litura* after treatment with pyriproxyfen, and we examined the effects of the hormone analog on fecundity, ovarian development, and the expression of *Vg*, *USP*, *HR3*, and *EcR* in *S*. *litura*. Our results suggest that the topical applications of a high concentration of pyriproxyfen on newly emerged *S*. *litura* females negatively regulated their reproductive properties and could be used to efficiently control its population growth instead of pesticides.

## Materials and Methods

### Insects

Larvae of *S*. *litura* were obtained from Henan Jiyuan Baiyun Industry (Jiyuan, Henan, China), and were reared in wooden cages (45 × 55 × 55 cm) at 30°C ± 1°C and 40% relative humidity with a 14:10 h (light/dark) photoperiod. Cotton balls soaked in 20% honey were provided to the adults for nutrition. After two generations, the eggs were collected daily, and newly emerged virgin adults were collected for the experiment.

### Pyriproxyfen treatment

A solution containing 100 μg/μL pyriproxyfen (4-phenoxyphenyl [RS]-2-2-pyridyloxypropyl ether; Sigma-Aldrich, St. Louis, MO, USA) in acetone was prepared, and stored at –20°C. Newly emerged females (day 0) were treated topically by applying 1 μL of pyriproxyfen (20, 60, or 100 μg/μL)to the abdomen. The pupae (day 0) were treated with 1 μL of pyriproxyfen (0.05 or 10 μg/μL). The control insects were treated with 1 μL of acetone.

### Gas Chromatography (GC) analysis

JH III and 20E were purchased from Sigma-Aldrich. The methanol was obtained from SK Energy (Seoul, ROK). All of the reagents used for GC were HPLC grade. We used GC to measure the titers of 20E and JH III after we confirmed the peak time using GC-MS. After the pupae and adult females were weighed, they were transferred to 1.5-mL microcentrifuge tubes, and 1 mL of methanol was added to each tube. The samples were sonicated for 10 min, and cooled on ice for 10 min. The samples were centrifuged at 100 000 × *g* for 10 min at 4°C, and the supernatant was transferred to a new microcentrifuge tube. The sonication and centrifugation steps were repeated. The supernatants were pooled and dried by evaporation at 37°C. The dried residue was dissolved in 200 μL of methanol, and the levels of JH III and 20E in each sample were measured using a Shimadzu GC-2010 system (Kyoto, Japan), as described by Munyiri and Ishikawa [31]. A Varian SP-5 elastic quartz capillary column (Agilent Technologies, Santa Clara, CA, USA), 30 m × 0.25 mm (i.d.) in size with a film thickness of 0.25 μm, was used for analysis the separation. Nitrogen was used as the carrier gas, and hydrogen was used as the detector gas at sample flow rates of 40 mL/min, an air gas flow rate of 400 mL/min, and a sample volume of 3.0 μL. The column was heated at 50°C for 2 min. The temperature was raised to 100°C at a rate of 4°C/min. Then, the column was heated to 270°C at a rate of 15°C/min, and held at 270°C for 10 min. The injector and transfer lines were at 280°C.

### Quantitative reverse transcription and real-time polymerase chain reaction (qRT-PCR)

The fat body was dissected from female *S*. *litura* by gently removing the intestine and reproductive organs, as described by Nose *et al*. [32]. The samples were pooled in RNase-free tubes, and placed on ice. Total RNA was extracted from the pooled samples using Trizol reagent (Invitrogen, Carlsbad, CA, USA), and the purified RNA was treated with RNase-free DNase (Promega, Madison, WI, USA) to remove the genomic DNA. The concentration of the purified RNA was measured using a Nanodrop spectrophotometer (Thermo Scientific, Waltham, MA, USA).

The primers used for qRT-PCR (S1 Table) were designed using the Primer3 software. First-strand cDNA synthesis was performed using AMV reverse transcriptase (Takara-Bio, Shiga, Japan), dNTPs, and 1 μg of total RNA as the template in a final volume of 25 μL. The cDNA samples were diluted 2-fold in PCR grade water. The qRT-PCR was performed using 10 μL of SsoFast EvaGreen Supermix (Bio-Rad Laboratories, Hercules, CA, USA), 1 μL of cDNA, 1 μL of each forward and reverse primer (10 μM), and 7 μL of PCR water. All of the samples were analyzed in triplicate using a CFX 96 Real-time PCR Detection System (Bio-Rad Laboratories). Thermal cycling was performed at 95°C for 15 s, followed by 45 cycles of 95°C for 8 s and 62°C for 25 s. A melting curve analysis was performed to ensure the homogeneity of the product. The mean and standard errors were determined for each time point.

### Transmission electron microscopy (TEM)

TEM was performed to examine the effects of pyriproxyfen on the ultrastructures of follicle cells and oocytes. The ovaries were dissected from adult females and placed in phosphate-buffered saline (PBS, pH 7.4). Ovary development was divided into different stages, based on the development of oocyte and follicle cells. The ovaries were fixed in 2.5% glutaraldehyde for at least 4 h. After washing three times with PBS, the samples were post-fixed in 1% osmium tetroxide for 1 h. After washing, the samples were dehydrated and infiltrated before being embedded in paraffin. Semi-thin sections were prepared and stained using uranyl acetate and alkaline lead citrate for 15 min. TEM was performed using a JEM-1230 electron microscope.

### Fecundity measurement

Healthy adult females (day 0) were treated topically with pyriproxyfen. The females were individually mated and reared separately in 250-mL plastic cups. Cotton balls soaked in 25% honey were provided for nutrition. The number of eggs deposited by each female was counted each day. The days having more than 100 eggs/female oviposited were considered as the oviposition peak. The lifespan of the females was recorded, and the number of premature oocytes remaining in the ovarioles was counted after the female died naturally. An analysis of variance was performed to examine the significance of the differences in fecundity between the pyriproxyfen-treated insects and the control insects. Females receiving 1 μL of acetone were used as controls.

## Results

### JH and 20E positively regulated vitellogenesis in *S*. *litura*

**Fig 1:**
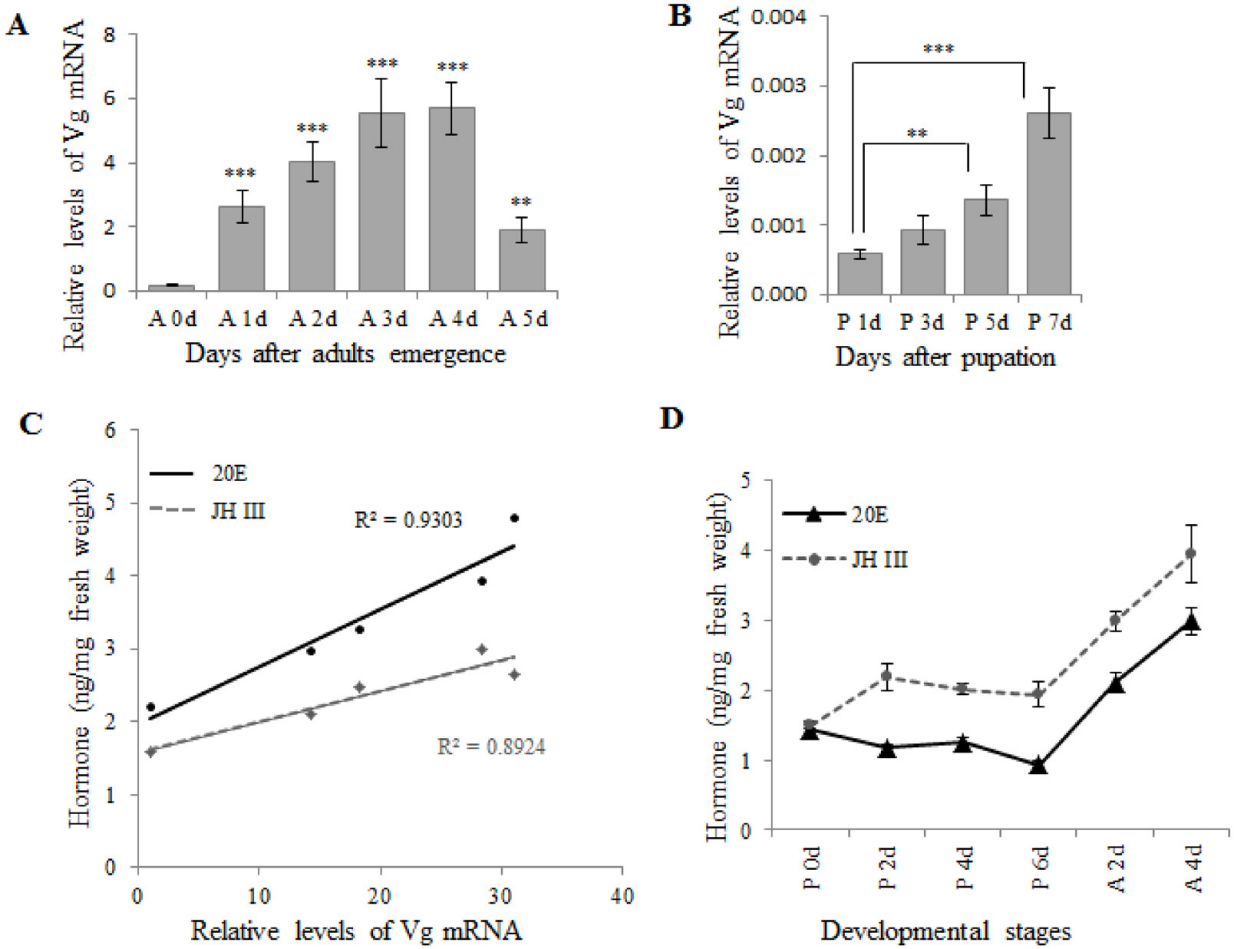
Correlation between hormone levels and *Vg* mRNA expression levels in *S*. *litura*. The level of *Vg* mRNA in female adults (A) and pupae (B). (C) Correlation analysis of hormone (JH III and 20E) and *Vg* expression levels. (D) The levels of JH III and 20E at different developmental stages. Total RNA was extracted from the fat body for qRT-PCR. The data represent at least three independent experiments with five female insects in each experiment, and are normalized relative to the level of β-actin mRNA. Vertical error bars indicate standard errors (**P ≤ 0.01; ***P ≤ 0.001).

In Lepidopterans, the patterns of hormonal regulation of vitellogenesis are differentiated between species. To determine whether JH and 20E have participated in regulating *Vg* gene transcription in *S*. *litura*, we performed qRT-PCR to analyze *Vg* mRNA levels, and we performed a GC analysis to quantify the amounts of JH III and 20E in treatment groups. In normal development, low-level *Vg* expression was observed during the pupal stage (Fig 1B). However, after molting, *Vg* expression gradually increased, reaching a peak on days 3 and 4 (Fig 1A). As we expected, in the pupal stage, the 20E level was stable, while the JH III level significantly increased on the second day and remained at a constant high level (JH III: *P* = 0.006; 20E: *P* = 0.102). Furthermore, the JH III and 20E levels increased rapidly at the onset of the adult stage (Fig 1D). The correlation analyses showed that JH III and 20E positively correlated with *Vg* expression (JH III: *R* ^2^ = 0.9303, *P* = 0.023; 20E: *R* ^2^ = 0.8924, *P* = 0.017; Fig 1C).

### Pyriproxyfen up-regulated *Vg*, *HR3*, and *USP* transcription

**Fig 2:**
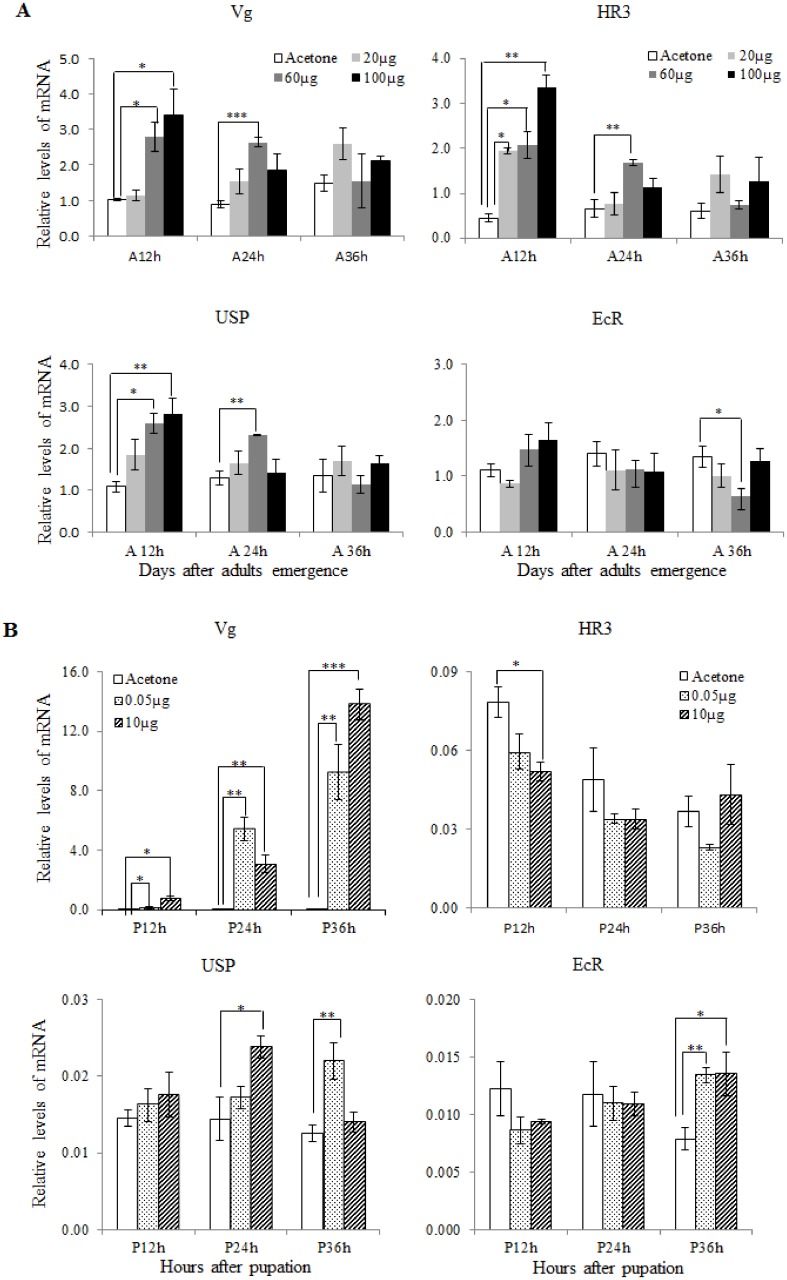
Topical application of pyriproxyfen increased *Vg*, *HR3*, *USP*, and *EcR* gene expression levels in (A) adults and (B) pupae of *S*. *litura*. Total RNA was extracted from the fat body at three time points. Data were collected from three independent experiments with five female insects in each experiment, and were normalized to the level of the β-actin mRNA. Vertical error bars indicate standard errors (*P ≤ 0.05; **P ≤ 0.01; ***P ≤ 0.001).

To understand the contribution of JH to vitellogenesis in *S*. *litura*, we topically applied pyriproxyfen to newly emerged females. A higher Vg gene expression level was detected in the treated females compared with the controls. The *Vg* gene expression level and three related hormone receptors were examined at 12, 24, and 36 h after treatment. The expression profile of *Vg* at 12 h showed that the *Vg* gene’s expression level increased as the pyriproxyfen dosage increased, suggesting that a higher JH titer is needed for Vg induction in *S*. *litura* (Fig 2A). Meanwhile, a similar expression profile was observed for two hormone receptors, *HR3* and *USP*, whereas the level of *EcR* expression was unaffected in pyriproxyfen-treated females (Fig 2A). We also found that pyriproxyfen induction of the *Vg*, *HR3*, and *USP* gene’s transcription was the most efficient at 12 h post-treatment, and gradually decreased during the subsequent 24-h period. Transcription of the Vg gene started in the pupal stage of *S*. *litura*, when they were more sensitive to the pyriproxyfen treatment, therefore, we used a low pyriproxyfen dose on the pupae. The Vg expression also increased significantly in pupae following pyriproxyfen treatment, and the change in *USP* expression was similar to that of *Vg* gene expression (Fig 2B). Interestingly, the hormone receptors *HR3* had an opposite expression profile compared with adult females in the treated group. The level of *EcR* expression was similar between the pyriproxyfen-treated and control insects during the first 24 h and was significantly up-regulated in both groups during the last 12 h of the observation period (Fig 2B).

### Pyriproxyfen reduces the number of eggs laid and shortens the female lifespan

**Fig 3:**
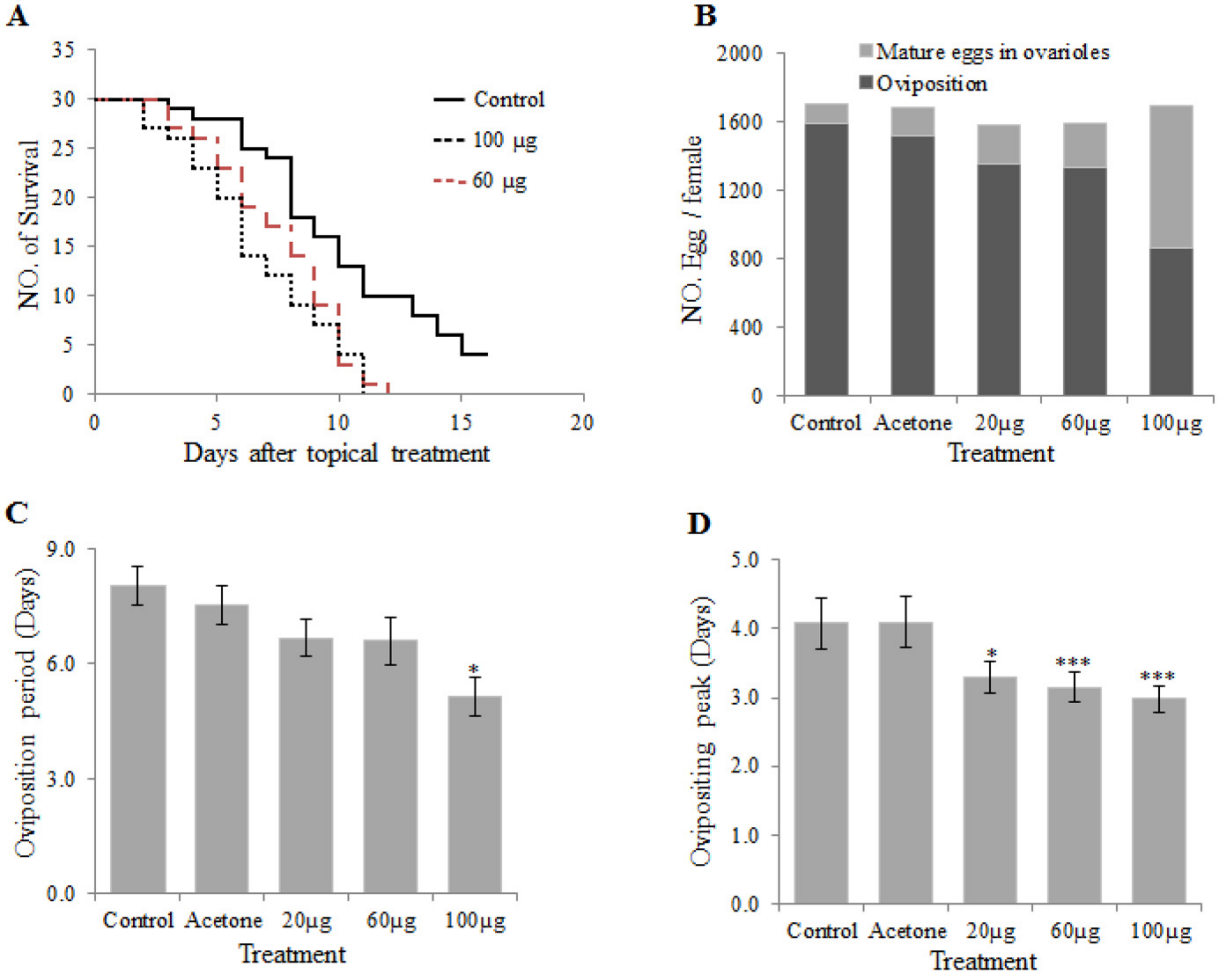
Effects of pyriproxyfen on adult reproduction and longevity. Newly emerged adult females were treated topically with pyriproxyfen, and survival (A), the number of eggs laid and the number of mature eggs in the ovarioles (B), the length of the oviposition period (C), and peak oviposition (D) were determined. Vertical error bars indicate standard errors, n = 10 to 30. (*P ≤ 0.05 and ***P ≤ 0.001).

To assess the effects of pyriproxyfen on lifespan and egg production, newly emerged adult females (day 0) were treated with 20, 60, or 100 μg of pyriproxyfen, and the number of eggs laid was recorded each day over the course of the female’s lifespan (S2 Table). The results on lifespan showed that 20 μg of pyriproxyfen did not influence the females’ longevity (S1 Fig). However, a topical application of 60 or 100 μg of pyriproxyfen significantly shortened the females’ longevity, such that insects treated with 100 μg of pyriproxyfen had a lifespan of approximately half that of the controls (Fig 3A). Therefore, although pyriproxyfen up-regulated the expression of *Vg*, *HR3*, and *USP* in adult females (Fig 2A), the number of eggs laid and the oviposition period were significantly reduced (Fig 3B and 3C). In addition, we observed that the peak oviposition period was progressively shortened with increasing dosages of pyriproxyfen (Fig 3D). After the adult female died, the ovary was dissected and the number of mature eggs remaining in the ovarioles was recorded. We found that the pyriproxyfen treatment significantly increased the number of mature eggs remaining in the ovarioles after the moth died (*df* = 4, *P* < 0.001), whereas the total number of eggs did not change (Fig 3B).

### Pyriproxyfen stimulated yolk deposition in oocytes

**Fig 4:**
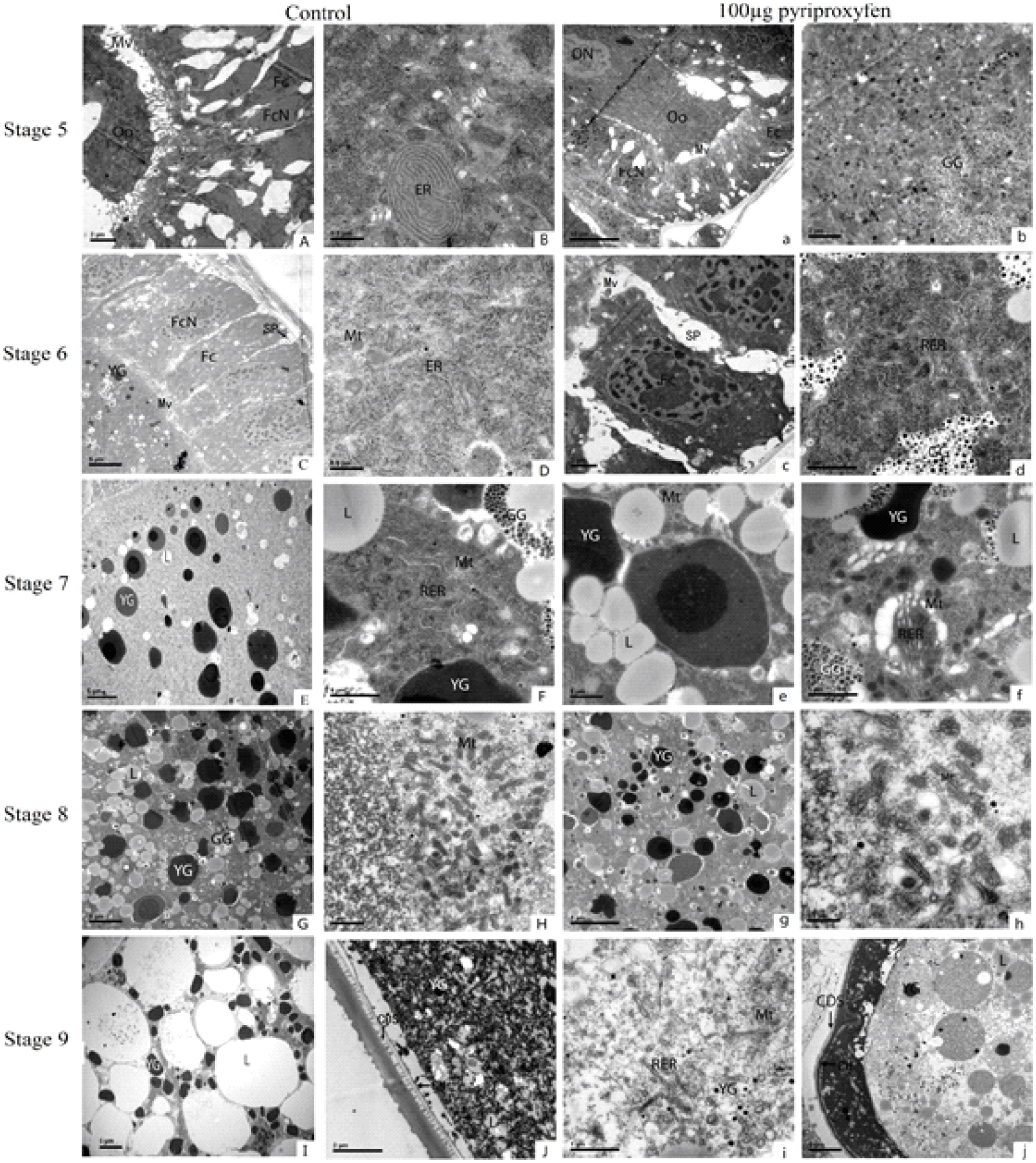
Effects of pyriproxyfen treatment on the ultrastructure of follicle cells and oocytes in the ovary. The transmission electron micrographs (TEM) images of the pyriproxyfen-treated and control females were compared. The first and second columns represent the TEM of the follicle cells and oocytes of control female *S*. *litura*; the third and fourth columns represent the TEM of the follicle cells and oocytes of pyriproxyfen-treated female *S*. *litura*. The fifth line represents the developed eggs. The oocytes (Oo) in the stage 5 of the controls were entirely surrounded by follicle cells (Fc) with numerous microvilli (Mv) (A), whereas fewer microvilli were observed in the ovaries of the pyriproxyfen-treated insects (a). FcN = the follicular epithelium cell nucleus. ON = oocyte nucleus. The endoplasmic reticulum (ER) can be observed in the control group (B), and yolk granules (YG) and glycogenosomes (GG) can be observed in pyriproxyfen-treated female *S*. *litura* (b). Follicle cells (Fc) and oocytes of control and pyriproxyfen-treated female in stage 6 (C, c), spaces appear between follicle cells. The pyriproxyfen treatment increased the amount of ER or rough ER (RER), the number of mitochondria (Mt), and the accumulation of lipids (L) in stage 6 and stage 7 (D, E and F; d, e and f). In stage 8 and stage 9 oocytes reduced numbers of YG and Mt were observed (G, H and I; g, h and i), and a comb-like dentate structure was observed in the mature oocytes (stage 9) of both groups (J and j). Scale Bars showed on down-left of each Figure.

We next determined whether JH has participated in regulating the Vg uptake by developing oocytes. The newly emerged females were topically treated with 100 μg pyriproxyfen, and the ovarioles were dissected after 24 h exposure. The development of oocyte was divided into nine different stages in *Tribolium molitor* [33] and *T*. *castaneum* [34]. To examine the effects of pyriproxyfen on the morphology of the organelles within the egg chambers, we recorded the developmental stages of oocyte from newly formed egg chambers (stage 5) to mature oocyte stage (stage 9) following the nomenclature used by Ullmann [33] and Parthasarathy *et al* [34]. The TEM images of the pyriproxyfen-treated and control females were compared (Fig 4). In the stage 5 (newly formed egg chambers) of the controls, the oocyte was entirely surrounded by follicle cells, with numerous microvilli projecting from the follicle cells onto the oocytes (Fig 4A), whereas fewer microvilli were observed in the ovaries of the pyriproxyfen-treated insects (Fig 4a). The endoplasmic reticulum could be observed in control oocytes while it could not be seen in the pyriproxyfen-treated ones. Additionally, large amounts of glycogenosomes (GGs) were found in the pyriproxyfen-treated oocytes (Fig 4B and 4b). In stage 6 (early vitellogenesis), the follicle cells were arranged in line surrounded the oocytes in both conditions (Fig 4C and 4c). But more extensive rough endoplasmic reticulum (RER) and GGs appeared in pyriproxyfen-treated females than in control ones (Fig 4D and 4d). Pyriproxyfen treatment increased the amount and volume of lipids (L), the number of RER and mitochondria (Mt) in stage 7 (midvitellogenesis) (Fig 4E, 4F, 4e and 4f). In this stage, the metabolism in the pyriproxyfen-treated oocytes seems more active than in the control ones with respect to changes in RER, Mt and GG in pyriproxyfen-treated insects, which might have resulted from the increase in *Vg* gene expression caused by pyriproxyfen. However, in stage 8 (late vitellogenesis), the amount of YG and L in pyriproxyfen-treated insects was less than those in the controls (Fig 4G and 4g). In the mature oocyte (stage 9), the amount and volume of L in control insects increased and the number of YGs in pyriproxyfen-treated insects decreased (Fig 4I and 4i). No significant effects on egg morphology were observed in the mature oocytes of females treated with pyriproxyfen, with both groups producing the typical cigar-shaped eggs (Fig 4J and 4j). However, the YG level was reduced in the mature oocytes of females treated with pyriproxyfen relative to that observed in the control oocytes (Fig 4j).

## Discussion

In our current study, we analyzed the effects of pyriproxyfen on the female reproductive system when it was topically applied to *S*. *litura* pupae or adults. Lepidopteran species can be divided into four groups based on differences in reproductive traits and development [12]. The JH and 20E hormone signaling pathway is may participate in the *Vg* transcriptional regulation of *S*. *frugiperda*, while only JH is crucial for Vg deposition in developing oocytes [15]. Because *S*. *litura* belongs to noctuidae, and we observed a positive correlation between the JH III and 20E levels and *Vg* gene expression during vitellogenesis, we speculated that the expression of the Vg gene might be regulated by both JH and 20E in *S*. *litura*.

Information regarding the effects of JHAs on reproduction in moths is scant, and most previous studies focused on the effects of JHAs on larvae and pupae [10, 35, 36]. In noctuid pests, like in *Heliothis virescens* and *Heliothis zea*, vitellogenesis is activated a few hours before or after eclosion [37, 38]. In *S*. *litura*, JH is an important hormone in regulating *Vg* gene expression, therefore, it is an ideal model for studying the effects of JH on female reproduction. Pyriproxyfen, a JHA, inhibits insect development throughout its life cycle. To examine its effects on vitellogenesis in *S*. *litura*, we treated the newly emerged female adults (day 0) with 20, 60, and 100 μg of pyriproxyfen. Our results showed that the effects of pyriproxyfen on *Vg* gene expression were dose-dependent, with a higher pyriproxyfen dosage resulting in a higher level of *Vg* expression (Fig 2). However, the effects of pyriproxyfen on *Vg* gene expression gradually decreased starting at 36 h post-treatment.

In hormonal signaling pathways, EcR and USP are important regulators of ecdysone activity [39–42]. HR3 is also an important downstream hormone receptor. In our current study, we found that the relative gene expression levels of *HR3* and *US*P were similar to that of *Vg*, suggesting that *USP* and *HR3* are directly involved in vitellogenesis in *S*. *litura*. However, we also found that the pyriproxyfen treatment di d not significantly affect *EcR* gene expression.

Pyriproxyfen has a negative effect on reproduction and lifespan in *S*. *litura*. Topical applications of pyriproxyfen on adult female did not change the number of mature eggs, but reduced egg laying in a dose-dependent manner. The data suggested that pyriproxyfen may inhibit embryogenesis at the early stage of embryo development, leading to a reduction of egg laying amount. This finding is consistent with those of recent studies on the influence of pyriproxyfen on fecundity and reproduction in *Stomoxys calcitrans* and *Monomorium pharaonis* [43, 44]. In *S*. *litura*, peak oviposition was delayed and lifespan was shortened in pyriproxyfen-treated females in a dose-dependent manner.

The role of JH in regulating Vg uptake has been well studied in *Rhodnius prolixus*, where JH assists epithelial patency by causing iso-osmotic shrinkage of the follicle cells [45] and may enhance endocytotic uptake by increasing the number of Vg receptors [46]. Studies in other insects, such as *S*. *frugiperda* and *Drosophila melanogaster*, JH primarily regulates Vg uptake into the eggs [15, 47]. Our TEM analysis of the oocytes of female *S*. *litura* treated with 100 μg of pyriproxyfen revealed significant changes in oocyte development. The RER and Mt are important organelles in protein synthesis and metabolism. We found that pyriproxyfen increased the amount of RER and the number of Mt in the oocytes of pyriproxyfen-treated females, which is consistent with increased *Vg* expression following pyriproxyfen treatment. We also observed more YG in the oocytes of pyriproxyfen-treated females (Fig 4). The data suggest that pyriproxyfen increased the uptake of Vg by developing oocytes, but whether similar mechanisms exist in *S*. *litura* needs further investigation.

Our findings indicate that high doses of pyriproxyfen may damage the reproductive system of female *S*. *litura*, resulting in the reduced viability of their eggs. In addition, treatments with the JHAs, juvenoid RO8-9801 and methoprene, completely inhibited yolk absorption by oocytes in *Blattella germanica*, and formed empty cavities and autophagic vacuoles in oocytes. JHAs cause protein degradation and the degeneration of the nuclei in oocytes, thereby rendering the eggs infertile [48–50]. Although pyriproxyfen applications could benefit the Vg deposition in the early stage of oocytes, they also damage the oocyte during later stages.

Oosorption is a phenomenon in which developing oocytes are resorbed in the ovary in response to internal and/or environmental factors, and it is a specific reproductive strategy that conserves resources and insures reproductive success [51]. During the pre-vitellogenic resting stage after initial ovarian maturation is complete [52], JH synthesis rates slowly decline from their peak, while ovarian follicles are resorbed through an apoptosis-like mechanism in the mosquito [53]. Resorption during this time is dependent on the quality of nutrition obtained through sugar feeding as well as JH [54]. JH analogs are known to inhibit the production of JH by the corpora allata, and allatostatin may be an effector through which fenoxycarb inhibits JH biosynthesis [55]. In our results, although pyriproxyfen up-regulated the expression of Vg gene in adult females (Fig 2A), the number of eggs laid and the oviposition period were significantly reduced (Fig 3B and 3C). The high titer of pyriproxyfen may inhibit the production of JH and interrupt the uptake of vitellogenin by oocytes, resulting in oosorption.

## Conclusions

The common cutworm, *S*. *litura*, is a polyphagous pest of numerous agricultural ecosystems that contributes to ecological damage and economic losses worldwide. Insecticides remain the primary means of controlling *S*. *litura*. However, pesticides pollute the environment and can affect human health. Therefore, alternative approaches, such as RNA interference or exogenous hormone analogs, are needed for controlling *S*. *litura*. We analyzed the effects of various doses of pyriproxyfen at different developmental stages on reproduction in *S*. *litura*. We found that pyriproxyfen up-regulated the expression of *Vg*, *USP*, and *HR3*, but did not affect the expression of *EcR*. Days with high egg laying rates (more than 100 eggs per day) progressively decreased with increasing pyriproxyfen doses, and the lifespan and oviposition period were shortened. Although the number of mature eggs was similar between the control and pyriproxyfen-treated females (approximately 1,800 eggs per female), we found that pyriproxyfen significantly increased the number of mature eggs that remained in the ovarioles after the moth died compared with in the controls. The TEM images showed that the primary eggs of pyriproxyfen-treated females had more extensive RER and an increased number of Mt, suggesting that pyriproxyfen increases metabolism in oocytes. Our results suggest that the topical application of pyriproxyfen on newly emerged females can efficiently control reproduction in *S*. *litura* and may represent an alternative to the use of toxic chemicals for controlling this important agricultural pest.

## Supporting Information

S1 Fig No significant change in longevity was observed after treatment with 20 μg of pyriproxyfen. (PDF)

S1 Table Primer sequences for qRT-PCR. (PDF)

S2 Table Effects of pyriproxifen on daily oviposition in *S. litura*. (PDF)
