# Supplementary figures and images for: Effects of Pyriproxyfen on Female Reproduction in the Common Cutworm, Spodoptera litura (F.) (Lepidoptera: Noctuidae)
Source: PLoS One. 2015 Oct 7;10(10):e0138171. doi: 10.1371/journal.pone.0138171 (PMC4596617; doi:10.1371/journal.pone.0138171)

**S1 Fig.**

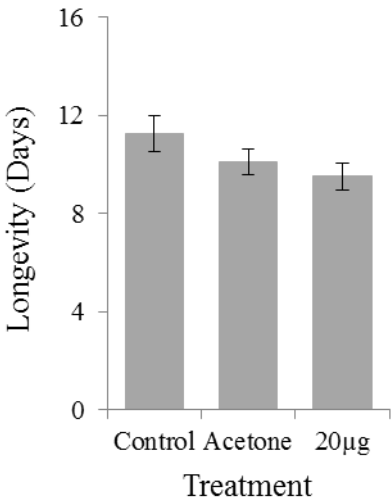

Supplement: S1 Fig — (PDF) [file pone.0138171.s001.pdf]
