# Supplementary material for: Effects of Pyriproxyfen on Female Reproduction in the Common Cutworm, Spodoptera litura (F.) (Lepidoptera: Noctuidae)
Source: PLoS One. 2015 Oct 7;10(10):e0138171. doi: 10.1371/journal.pone.0138171 (PMC4596617; doi:10.1371/journal.pone.0138171)
Supplement: S1 Table — (PDF) [file pone.0138171.s002.pdf]

S1 Table. Primer sequences for qRT-PCR

GENE	Forward primer	Reverse primer	GenBank Accession
EcR	GCACAATGGAGCAACAGCAGC	AGACCCTTAGCGAATTCGAC	Q730733
USP	CATCCTGGAACGAACTGCTA	GAGTTGTGGTGGCGATGTGGAC	EU180022
HR3	CGAGTTGGCCATTGTGAGAC	TCGCCGCCTCAAATATACC	
Vg	CACTCCGCTGTATCTCGCCTTCT	CGGGGGCATTGTCTTGTTCCAC	EU095334
β-Actin	GGCCAACAGGGAGAAGATGAC	CCGGAGTCCAGGACGATAC	DQ494753
