# Supplementary material for: Effects of Pyriproxyfen on Female Reproduction in the Common Cutworm, Spodoptera litura (F.) (Lepidoptera: Noctuidae)
Source: PLoS One. 2015 Oct 7;10(10):e0138171. doi: 10.1371/journal.pone.0138171 (PMC4596617; doi:10.1371/journal.pone.0138171)
Supplement: S2 Table — (PDF) [file pone.0138171.s003.pdf]

S2 Table. Effect of pyriproxifen on daily ovisposition in *S. litura*

	Control	Acetone	20μg	60μg	100μg
1stA	83±30	122±39	72±23	40±26	35±20
2ndA	323±63	340±71	334±67	491±68	290±55
3rdA	297±53	310±55	385±44	349±39	271±40
4thA	299±48	232±41	248±27	230±33	170±27
5thA	201±25	155±26	153±23	147±27	86±20
6thA	157±19	163±27	79±12	77±16	62±15
7thA	142±24	119±19	49±11	40±11	43±15
8thA	77±17	90±18	29±9	31±9	18±6
9thA	56±17	59±15	31±14	23±8	14±6
10thA	37±12	39±17	35±13	12±4	6±6
11thA	22±10	38±28	19±10	19±12	5±5
